# Prevalence of anemia and its associated factors among married women in 19 sub-Saharan African countries

**DOI:** 10.1186/s13690-021-00733-x

**Published:** 2021-11-29

**Authors:** Betregiorgis Zegeye, Felix Emeka Anyiam, Bright Opoku Ahinkorah, Edward Kwabena Ameyaw, Eugene Budu, Abdul-Aziz Seidu, Sanni Yaya

**Affiliations:** 1 HaSET Maternal and Child Health Research Program, Addis Ababa, Ethiopia; 2 Centre for Health and Development, University of Port Harcourt, Port Harcourt, Nigeria; 3 School of Public Health, University of Technology Sydney, Sydney, Australia; 4 Centre For Gender and Advocacy, Takoradi Technical University, Takoradi, Ghana; 5 School of International Development and Global Studies, University of Ottawa, Ottawa, Ontario Canada; 6 The George Institute for Global Health, Imperial College London, London, UK

**Keywords:** Anemia, Factors, Sub-Saharan Africa, Married women, DHS, Global health

## Abstract

**Background:**

Sub-Saharan Africa (SSA) remains the region with the highest burden of anemia globally. Since anemia has both health and non-health-related consequences, its reduction is one of the Sustainable Development Goals. Therefore, this study aimed to examine the prevalence of anemia and its associated factors among married women in SSA.

**Methods:**

Using Stata version-14 software, the analysis was done on 89,029 married women from the Demographic and Health Surveys of 19 countries in SSA. Pearson Chi-Square test and Binary logistic regression analyses were used to examine the factors associated with anemia. The results were presented using adjusted Odds Ratio (aOR) at a 95% Confidence Interval (CI). A *p*-value less than or equal to 0.05 (*p* ≤ 0.05) was considered statistically significant.

**Results:**

The pooled analysis showed that 49.7% of married women were anemic. Of these, 1.04% and 15.05% were severely and moderately anemic respectively, and the rest 33.61% were mildly anemic. Husband education (primary school-aOR = 0.84, 95% CI; 0.71–0.99), wealth index (middle-aOR = 0.81, 95% CI; 0.68–0.96, richer-aOR = 0.69, 95% CI; 0.57–0.84, richest-aOR = 0.68, 95% CI; 0.51–0.91), modern contraceptive use (yes-aOR = 0.68, 95% CI; 0.56–0.81) and religion (Muslim-aOR = 1.27, 95% CI; 1.11–1.46, others-aOR = 0.73, 95% CI; 0.59–0.90) were factors associated with anemia among married women.

**Conclusion:**

The findings show that nearly half of the married women are affected by anemia. Enhancing partners’ educational levels, and economic empowerment of women, strengthening family planning services, and working with religious leaders to reduce the perception and religious beliefs related to food restrictions can be the main focus to reduce the burden of anemia among married women in SSA.

## Background

Anemia is one of the common and major public health and nutritional problems globally [1–3]. The World Health Organization (WHO) defines anemia as a condition in which the number of red blood cells (oxygen caring capacity) is not adequate to meet the body’s physiologic needs [1, 2, 4]. Globally, anemia affects about two billion people, or one-third of the adult population lives [5–7]. Of these, half a billion are reproductive age women (15–49 years) [8]. According to the 2016 World Bank evidence, 33% of women in the reproductive-age groups were anemic worldwide [9]. In low and middle-income countries, nearly 35.4% of reproductive age women were anemic as of 2016 [9]. However, the prevalence of anemia in sub-Saharan African countries increased  to 39% in the same period [9].

Women are among the vulnerable groups mainly due to the experience of menstruation, and pregnancy and childbirth-related blood loss [2]. Anemia among reproductive-age women can be caused by nutritional and non-nutritional causes [10–14]. Anemia leads to dizziness, and fatigue, poor health, and productivity among the general population [15]. The consequence of anemia is high especially among pregnant women because it increases the chances of developing complications for the women and the neonate that could include miscarriage, intrauterine fetal death, preterm delivery, low birth weight, and mortality [16, 17]. Anemia has non-health-related consequences such as high health-care expenditures, decreasing income, and related socio-economic problems among families and communities have also been documented [18].

Hence, emphasis on the reduction of anemia prevalence among reproductive-age women is essential and has a multidimensional and intergenerational importance in enhancing healthier pregnancy, women’s and child health, school performance, productivity, and development in general [18–20]. Anemia reduction is one of the Sustainable Development Goals (SDGs) [21] and the World Health Assembly Nutrition Targets for 2025 [22]. Progress in the reduction of anemia has been seen globally and in African countries, however, reduction in anemia prevalence is not on track as targeted by World Health Assembly, i.e. 50% reduction among reproductive-aged women by 2025 [22].

There are few studies in African countries that show that individual and community level factors were linked with anemia among reproductive-age women [23–28]. However, these studies are either single country [23–27] or in East African countries alone [28]. Therefore, limited evidence on anemia at the sub-regional level is lacking. Hence, we examined the prevalence and factors associated with anemia among married women in sub-Saharan Africa (SSA). The findings from the present study could help policymakers and programme implementers to review the implementation of anemia reduction strategies and interventions and to speed-up anemia reduction targets by the World Health Assembly [22] and SDGs [21] at the national and regional levels.

## Methods

### Data source

We used data from the Demographic and Health Surveys (DHSs) of 19 sub-Saharan African countries, that were conducted between 2010 and 2019. DHS is a nationally representative survey that is carried out across several low- and middle-income countries and focus on data collection on several demographic and health indicators including anemia [29]. It is carried out with the financial and technical support of the United States Aid for International Development (USAID) and Inner City Fund (ICF) International respectively [30].

DHSs have applied a two-stage stratified cluster sampling technique. In the first stage, Enumeration Areas (EAs) were selected using Probability Proportional to Size (PPS) and in the second stage, fixed number of households (usually 25–30 households) are selected using systematic sampling technique, from the selected EA [31]. We used the individual recode (IR) file for this analysis and a total of 89,029 married women were included for the analysis (see Table 1). The dataset is freely available for download at https://dhsprogram.com/data/available-datasets.cfm. We followed the guidelines for Strengthening Observational studies in Epidemiology (STROBE) during the preparation of this manuscript [32].

**Table 1:** Year of the survey and weighted sample of each studied country

Country	Year of survey	Weighted sample
Burkina Faso	2010	6570
Benin	2017/18	5654
Burundi	2016/17	4703
Congo Democratic Republic	2013/14	6155
Cote d’Ivoire	2011/12	3006
Cameroon	2018/19	3786
Ethiopia	2016	9220
Gabon	2012	3140
Ghana	2014	2745
Gambia	2013	3119
Guinea	2018	3767
Mali	2018	4060
Malawi	2015/16	5232
Rwanda	2014/15	3384
Sierra Leone	2019	4602
Togo	2013/14	3210
Uganda	2016	3745
Zambia	2018/19	7361
Zimbabwe	2015	5570
**Total**		**89,029**

### Study variables

#### Outcome variable

The outcome variable for this study was the prevalence of anemia among married women. For non-pregnant women, anemia was defined as hemoglobin count less than 12.0 g per deciliter (g/dl) and less than 11.0 g/dl for pregnant women. Mild anemia for non-pregnant women was defined as hemoglobin count between 10.0 and 11.9 g/dl and for pregnant women between 10.0 and 10.9 g/dl. Moderate anemia for both pregnant and non-pregnant women was defined as hemoglobin count between 7.0 and 9.9 g/dl. Severe anemia was defined as a hemoglobin count less than 7.0 g/dl [8, 18].

#### Explanatory variables

With reference to previous studies [11–15, 23–28], we included the following explanatory variables due to their association with anemia among women. These were women’s age in years (15–19, 20–24, 25–30, 31–34, 35–40, 41–44, 45–49), women’s educational level (no formal education, primary school, secondary school and higher), husband’s educational level (no formal education, primary school, secondary school, higher), women’s occupation (not working, professional or technical or managerial, agricultural, manual, others) and wealth index (poorest, poor, middle, rich, richest). Additionally, we included place of residence (urban, rural), reading newspaper (no, yes), listening to radio (no, yes), watching television (no, yes), sex of household head (male, female), decision making capacity (no, yes), wife beating attitude (accept/justify, disagree/not justify), parity (zero, 1–2, 3–4, 5+), family size (< 5, 5+), barriers to healthcare access (no, yes), improved sanitation (no, yes), religion (Christian, Muslim, others) and contraceptive use (no, yes).

### Statistical analyses

The analysis for this study was carried out using the following steps. First, descriptive analyses such as frequency distribution and percentages were used to show the prevalence of anemia. Pearsons Chi-Square test was used to test for proportional difference between the explanatory variables and anemia and Bivariate logistic regression analysis was used to examine the crude odds of each explanatory variable with the outcome variable. Multicollinearity test was carried out to examine whether or not collinearity among the explanatory variables existed using Variance Inflation Factor (VIF) and we confirmed that there was no evidence of collinearity (VIF Mean = 1.96, VIF Min = 1.08, Max VIF = 3.15). Finally, all statistically significant explanatory variables in the bivariate logistic regression were entered into a Multivariate logistic regression model. The adequacy of the model was checked by Hosmer-Lemeshow and there was confirmation that the model was a good fit (*P*-value = 0.9497). The results were presented using crude odds ratio (cOR) and adjusted odds ratio (aOR) with a 95% Confidence Interval (CI). A *P*-value less than or equal to 0.05 (*p* ≤ 0.05) was considered statistically significant. To take care of the complex nature of the DHS’s data, we used the “svyset” command during the analysis, and all three design elements such as weight, cluster, and strata were taken into consideration.

### Ethical clearance

We used secondary data that are publicly available (https://dhsprogram.com/data/available-datasets.cfm). Ethical procedures are the responsibility of institutions that funded, commissioned, and managed the surveys, and so further ethical clearance was not required. ICF international approved all the DHS surveys and ensured that the study follows the U.S. Department of Health and Human Services rules for respecting the rights of human subjects. For more details related to ethical issues, readers can visit http://goo.gl/ny8T6X.

## Results

### Background characteristics of the sampled population

In total, 89,029 married women were included in the analysis. Nearly 7.7% of the respondents were young women aged between 15 and 19 years, and the majority were 25–29 years (20.45%). Over half (78.8%) of the respondents were rural residents. About 82% of respondents and 79.4% of their husbands had no formal education. Regarding women empowerment, about 88% of married women had no decision-making power for at least one of the three decision-making parameters; their own health, to purchase large household expenses, and to visit families or relatives. Approximately 46.3% of married women accepted or justified wife-beating for at least one of the five reasons; burning food, arguing with husband, going out without telling their husband, neglecting children, and refusing sexual intercourse.

### Prevalence of anemia among women in sub-Saharan Africa

**Fig. 1:**
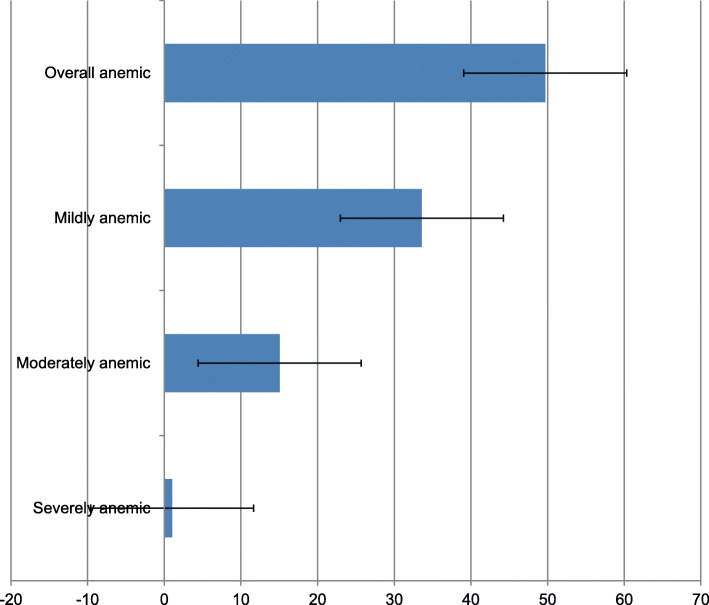
Prevalence of anemia among married women in the reproductive age groups (15–49 years): Evidence from pooled results of DHSs of 19 SSA countries

As shown in Fig. 1, the pooled result shows that 49.7% of married women in the reproductive age groups were anemic. Of these, 1.04% and 15.05% were severely and moderately anemic respectively, and the rest 33.61% were mildly anemic (Fig. 1).

**Fig. 2:**
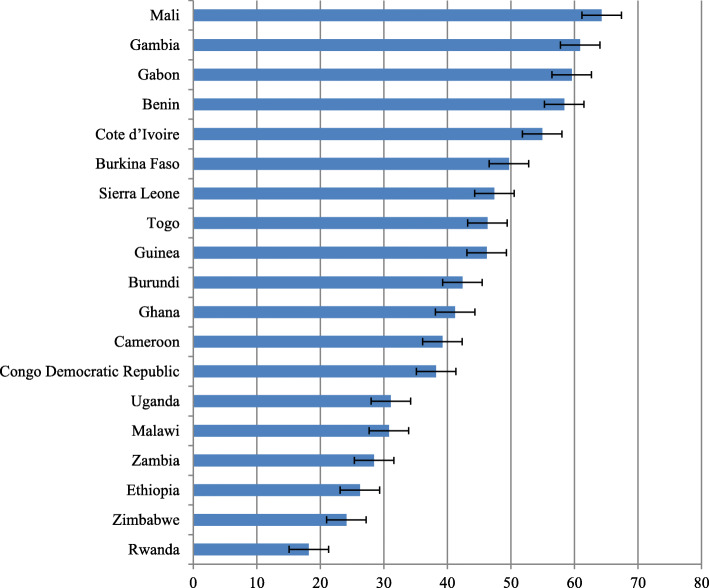
Prevalence of anemia among married women in the reproductive age groups (15–49 years) across 19 sub-Saharan African countries

Regarding the prevalence of anemia across countries, the study shows that the highest prevalences of anemia were in Mali (64.3%), Gambia (60.9%), Gabon (59.6%), and Benin (58.4%). On the other hand, lower prevalences of anemia was reported in Rwanda (18.2%), Zimbabwe (24.1%), Ethiopia (26.3%), and Zambia (28.5%) respectively (Fig. 2).

### Distribution of prevalence of anemia across explanatory variables

As shown in Table 2, anemia prevalence varied across socio-demographic and socio-economic variables. For instance, about 48.4% of married women who had not attained formal education were anemic, while 30% of married women who attended higher schools were anemic. Similarly, nearly half (49.4%) of women whose husbands had no formal education were anemic and it lowered to 34.7% among married women whose husbands had higher education. The prevalence of anemia also varied from approximately 45.5% to 34.2% among married women in the poorest and richest households respectively. Additionally, anemia prevalence varied based on religious affiliation. For instance, more than half (51.1%) of married Muslim women were anemic, while the prevalence lowered to about 35.3% among married women who were Christians (Table 2).

**Table 2:** Background characteristics of respondents and distribution of anemia across explanatory variables: Evidence from DHSs of 19 sub-Saharan African countries

Variables	Frequency (Weighted %)	Anemia		Chi-square, ***P***-value
		No, Frequency (Weighted %)	Yes, Frequency (Weighted %)	
**Age in years**				χ2 = 108.13, *p* < 0.001
15–19	10,811 (7.69)	2992 (52.81)	2674 (47.19)	
20–24	29,487 (19.80)	9183 (58.70)	6462 (41.30)	
25–29	35,814 (20.45)	11,310 (59.35)	7746 (40.65)	
30–34	31,562 (18.09)	10,079 (60.22)	6658 (39.78)	
35–39	26,553 (13.99)	8301 (58.74)	5831 (41.26)	
40–44	18,935 (11.39)	5917 (58.55)	4189 (41.45)	
45–49	14,431 (8.60)	4639 (60.35)	3048 (39.65)	
**Women’s educational level**				χ2 = 1400, *p* < 0.001
No formal education	71,213 (82.01)	19,126 (51.57)	17,959 (48.43)	
Primary school	55,720 (11.12)	18,580 (64.08)	10,414 (35.92)	
Secondary school	35,524 (6.06)	12,764 (63.31)	7398 (36.69)	
Higher	5136 (0.8)	1951 (69.98)	837 (30.02)	
**Husband's educational level**				χ2 = 1500, *p* < 0.001
No formal education	63,323 (79.43)	16,577 (50.58)	16,200 (49.42)	
Primary school	46,825 (12.03)	15,730 (64.81)	8542 (35.19)	
Secondary school	45,673 (7.09)	15,949 (62.39)	9616 (37.61)	
Higher	11,629 (1.45)	4128 (65.35)	2189 (34.65)	
**Women's occupation**				χ2 = 122.76, *p* < 0.001
Not working	42,230 (18.49)	14,602 (57.90)	10,616 (42.10)	
Professional or technical or managerial	5770 (1.12)	1917 (66.45)	968 (33.55)	
Agricultural	66,301 (49.72)	19,418 (58.72)	13,653 (41.28)	
Manual	11,985 (6.98)	3560 (63.04)	2087 (36.96)	
Others	41,229 (23.68)	12,906 (58.23)	9256 (41.77)	
**Wealth index**				χ2 = 567.88, *p* < 0.001
Poorest	37,255 (18.68)	11,192 (54.54)	9330 (45.46)	
Poor	34,176 (20.48)	10,183 (56.46)	7852 (43.54)	
Middle	32,652 (20.63)	9874 (57.99)	7152 (42.01)	
Rich	31,465 (20.61)	10,119 (60.76)	6536 (39.24)	
Richest	32,045 (19.59)	11,053 (65.83)	5738 (34.17)	
**Place of residence**				χ2 = 49.70, *p* < 0.001
Urban	51,703 (21.18)	16,896 (60.60)	10,984 (39.40)	
Rural	115,890 (78.82)	35,525 (58.10)	25,624 (41.90)	
**Reading of newspaper**				χ2 = 22.99,, *p* < 0.001
No	12,602 (94.09)	3109 (50.23)	3081 (49.77)	
Yes	790 (5.91)	239 (62.89)	141 (37.11)	
**Listening to radio**				χ2 = 16.33,, *p* < 0.001
No	3826 (30.04)	887 (47.03)	999 (52.97)	
Yes	9566 (69.96)	2461 (52.54)	2223 (47.46)	
**Watching television**				χ2 = 23.31,, *p* < 0.001
No	9773 (73.18)	2365 (49.16)	2446 (50.84)	
Yes	3619 (26.82)	983 (55.88)	776 (44.12)	
**Sex of household head**				χ2 = 0.4130 *p* = 0.520
Male	142,558 (94.19)	43,724 (58.83)	30,594 (41.17)	
Female	25,632 (5.81)	8697 (59.12)	6014 (40.88)	
**Decision making capacity**				χ2 = 752.00, *p* < 0.001
No	96,244 (88.03)	26,704 (54.77)	22,051 (45.23)	
Yes	71,219 (11.97)	25,672 (63.86)	14,527 (36.14)	
**Wife beating attitude**				χ2 = 124.99, *p* < 0.001
Accept/justify	82,620 (46.33)	25,723 (57.06)	19,357 (42.94)	
Disagree/Not justify	84,903 (53.67)	26,678 (60.75)	17,237 (39.25)	
**Parity**				χ2 = 86.12, *p* < 0.001
Zero	10,882 (07.04)	3110 (55.32)	2512 (44.68)	
1–2	51,939 (27.75)	16,740 (60.44)	10,956 (39.56)	
3–4	47,235 (26.04)	14,985 (59.61)	10,154 (40.39)	
5+	57,537 (39.17)	17,586 (57.52)	12,986 (42.48)	
**Family size**				χ2 = 83.51, *p* < 0.001
< 5	52,070 (25.32)	17,266 (61.09)	10,997 (38.91)	
5+	115,523 (74.68)	35,155 (57.85)	25,611 (42.15)	
**Barriers to healthcare access**				χ2 = 162.73, *p* < 0.001
No	56,307 (20.01)	18,964 (61.79)	11,728 (38.21)	
Yes	111,204 (79.99)	33,443 (57.36)	24,859 (42.64)	
**Improved sanitation**				χ2 = 303.33, *p* < 0.001
No	88,343 (71.25)	27,212 (56.25)	21,167 (43.75)	
Yes	79,822 (28.75)	25,200 (62.01)	15,436 (37.99)	
**Religion**				χ2 = 2000, *p* < 0.001
Christian	101,456 (26.91)	35,626 (64.71)	19,432 (35.29)	
Muslim	58,004 (63.85)	14,321 (48.89)	14,974 (51.11)	
Others	8658 (9.24)	2453 (52.88)	2186 (47.12)	
**Contraceptive use**				χ2 = 2200, *p* < 0.001
No	124,843 (84.98)	35,318 (54.21)	29,829 (45.79)	
Yes	42,750 (15.02)	17,103 (71.61)	6779 (28.39)	

*Ref* references, *cOR* crude Odd Ratio, *aOR* adjusted Odd Ratio, * significant at *p <* 0.05, ** significant at *p* < 0.01, *** significant at *p* < 0.001.

### Factors associated with anemia among women in sub-Saharan Africa

#### Bivariate logistic regression results

As shown in Table 3, several factors such as women’s age, women’s educational level, husbands’ educational level, women’s occupation, wealth index, reading of newspaper, listening to radio, watching television, place of residence, improved sanitation, contraceptive use, and religion were associated with anemia among married women in the bivariate logistic regression model (Table 3).

**Table 3:** Bivariate logistic regression results for factors associated with anemia among married women: Evidence from DHSs of 19 sub-Saharan African countries

Variables	cOR[95% CI]	***P***-value
**Age in years**		
15–19	Ref	
20–24	0.79 (0.63–0.99)*	**0.047**
25–29	0.79 (0.63–0.99)*	**0.041**
30–34	0.86 (0.68–1.08)	0.197
35–39	0.79 (0.64–0.98)*	**0.039**
40–44	0.86 (0.67–1.11)	0.277
45–49	0.73 (0.56–0.95)*	**0.021**
**Women’s educational level**		
No formal education	Ref	
Primary school	0.72 (0.60–0.86)***	***p*** **< 0.001**
Secondary school	0.49 (0.38–0.63)***	***p*** **< 0.001**
Higher	0.39 (0.19–0.81)*	**0.012**
**Husband's educational level**		
No formal education	Ref	
Primary school	0.66 (0.56–0.79)***	***p*** **< 0.001**
Secondary school	0.53 (0.42–0.68)***	***p*** **< 0.001**
Higher	0.45 (0.21–0.95)*	**0.037**
**Women's occupation**		
Not working	Ref	
Professional or technical or managerial	0.28 (0.15–0.52)***	***p*** **< 0.001**
Agricultural	0.88 (0.74–1.06)	0.213
Manual	0.62 (0.48–0.82)**	**0.001**
Others	0.78 (0.64–0.95)*	**0.016**
**Wealth index**		
Poorest	Ref	
Poor	0.92 (0.77–1.11)	0.434
Middle	0.83 (0.70–0.99)*	**0.047**
Rich	0.67 (0.56–0.81)***	***p*** **< 0.001**
Richest	0.52 (0.42–0.64)***	***p*** **< 0.001**
**Reading of newspaper**		
No		
Yes	0.53 (0.39–0.73)***	***p*** **< 0.001**
**Listening to radio**		
No		
Yes	0.83 (0.72–0.94)**	**0.006**
**Watching television**		
No		
Yes	0.72 (0.63–0.83)***	***p*** **< 0.001**
**Place of residence**		
Urban	Ref	
Rural	1.55 (1.32–1.83)***	***p*** **< 0.001**
**Decision making**		
No	Ref	
Yes	0.90 (0.75–1.08)	0.268
**Wife beating attitude**		
Accept/justify	Ref	
Disagree/Not justify	1.03 (0.92–1.15)	0.553
**Family size**		
< 5	Ref	
5+	0.96 (0.84–1.09)	0.556
**Barriers to healthcare access**		
No	Ref	
Yes	1.14 (0.99–1.32)	0.060
**Improved sanitation**		
No	Ref	
Yes	0.65 (0.56–0.76)***	***p*** **< 0.001**
**Contraceptive use**		
No	Ref	
Yes	0.56 (0.47–0.67)***	***p*** **< 0.001**
**Religion**		
Christian	Ref	
Muslim	1.43 (1.25–1.64)***	***p*** **< 0.001**
Others	0.92 (0.75–1.13)	0.444
**Parity**		
Zero	Ref	
1–2	1.15 (0.90–1.46)	0.242
3–4	1.12 (0.87–1.43)	0.358
5+	1.16 (0.91–1.47)	0.225

*Ref* references, *cOR* crude Odd Ratio, *aOR* adjusted Odd Ratio, * significant at *p* < 0.05, ** significant at *p* < 0.01, *** significant at *p* < 0.001.

### Multivariate logistic regression results

We observed that husbands’ educational level, wealth index, contraceptive use, and religion were the main factors associated with anemia among married women. More specifically, the study shows lower odds of anemia among married women whose husbands had primary education (aOR = 0.84, 95% CI; 0.71–0.99) as compared to married women whose husbands had no formal education. Moreover, we found lower odds of anemia among married from the middle (aOR = 0.81, 95% CI; 0.68–0.96), richer (aOR = 0.69, 95% CI; 0.57–0.84), and richest (aOR = 0.68, 95% CI; 0.51–0.91) wealth index as compared to married women in the poorest wealth index. Again, the study shows lower odds of anemia among married women who were currently using modern contraceptives (aOR = 0.68, 95% CI; 0.56–0.81) as compared to married women who were not using. Finally, the study shows higher odds of anemia among married women who belonged to the Islamic faith (aOR = 1.27, 95% CI; 1.11–1.46) as compared to married women who belonged to the Christian faith. Lower odds of anemia occurred among married women who belonged to other religions (aOR = 0.73, 95% CI; 0.59–0.90) as compared to married women who proffered the Christian faith (Table 4).

**Table 4:** Multivariable logistic regression results for factors associated with anemia among married women: Evidence from the DHSs of 19 sub-Saharan African countries

Variables	aOR[95% CI]	***P***-value
**Age in years**		
15–19	Ref	
20–24	0.89 (0.71–1.12)	0.360
25–29	0.92 (0.73–1.16)	0.515
30–34	0.99 (0.78–1.25)	0.975
35–39	0.89 (0.71–1.12)	0.348
40–44	0.97 (0.74–1.27)	0.853
45–49	0.79 (0.60–1.04)	0.094
**Women’s educational level**		
No formal education	Ref	
Primary school	0.91 (0.76–1.10)	0.380
Secondary school	0.74 (0.53–1.03)	0.080
Higher	0.71 (0.36–1.40)	0.333
**Husband's educational level**		
No formal education	Ref	
Primary school	0.84 (0.71–0.99)*	**0.045**
Secondary school	0.87 (0.66–1.14)	0.314
Higher	0.95 (0.47–1.91)	0.895
**Women's occupation**		
Not working	Ref	
Professional or technical or managerial	0.55 (0.30–1.01)	0.055
Agricultural	0.82 (0.67–1.01)	0.074
Manual	0.76 (0.58–1.00)	0.053
Others	0.89 (0.72–1.08)	0.256
**Wealth index**		
Poorest	Ref	
Poor	0.90 (0.75–1.08)	0.286
Middle	0.81 (0.68–0.96)*	**0.020**
Rich	0.69 (0.57–0.84)***	**0.000**
Richest	0.68 (0.51–0.91)*	**0.011**
**Reading of newspaper**		
No	Ref	
Yes	1.09 (0.76–1.56)	0.614
**Listening to radio**		
No	Ref	
Yes	0.94 (0.82–1.07)	0.400
**Watching television**		
No	Ref	
Yes	0.99 (0.85–1.15)	0.925
**Place of residence**		
Urban	Ref	
Rural	1.07 (0.88–1.30)	0.477
**Improved sanitation**		
No	Ref	
Yes	0.92 (0.75–1.14)	0.478
**Contraceptive use**		
No	Ref	
Yes	0.68 (0.56–0.81)***	**0.000**
**Religion**		
Christian	Ref	
Muslim	1.27 (1.11–1.46)**	**0.001**
Others	0.73 (0.59–0.90)**	**0.003**

*Ref* references, *cOR* crude Odd Ratio, *aOR* adjusted Odd Ratio, * significant at *p* < 0.05, ** significant at *p* < 0.01, *** significant at *p* < 0.001.

## Discussion

This study examined the prevalence of anemia and its associated factors among married women in 19 countries in SSA. The pooled analysis shows 49.7% of married women in the studied countries were anemic. Of these, 1.04% and 15.05% were severely and moderately anemic respectively, and the rest 33.61% were mildly anemic. The prevalence from this study is higher as compared to those reported in a recent study in East Africa [28] that was 34% but slightly lowered as compared to the study in seven South and Southeast Asian Countries (52.5%) [33]. The difference in prevalence was probably due to differences in the target population as reported by prior studies (married women had higher odds of being anemic as compared to never-married women) [28]. Again, methodological variations applied in the two studies and differences in the number of countries involved in the analysis might explain the differences in the findings. Out of the 19 studied countries, the lowest prevalence of anemia was reported in Rwanda (18.2%) and the highest prevalence was in Mali (64.3%). The variation of anemia might be due to differences in socioeconomic and other factors linked to anemia across countries [28, 33].

Consistent with previous studies in Ethiopia [3], India [34], and in Port Blair, Andaman, and Nicobar Islands [35], we found that the odds of anemia among married women whose husbands were educated were lower as compared to married women whose husbands had no formal education. This could be due to better receptive capacities of advice from healthcare workers and other sources regarding the prevention of anemia [34]. Educated husbands might encourage their wives to use modern healthcare services such as contraceptives, antenatal and postnatal care that in turn reduce the odds of the prevalence of anemia [3, 36].

The present study showed lower odds of anemia among married women who were in higher socio-economic status. Previous studies in Rwanda [23, 26], Ethiopia [24], and Uganda [27] documented comparable findings. The plausible reason could be the association between socioeconomic status and intake of a healthy diet, lower chance of infection, and better access and utilization of healthcare services [37–39]. The individuals in higher socioeconomic class have higher capacities of purchasing sufficient and various foods that lead them to have a lower prevalence of anemia [28].

This study observed lower odds of anemia among married women who were currently using modern contraceptives as compared to married women who were not using a modern contraceptive. Consistent findings were reported in Ethiopia [25, 28], Rwanda [26], and 24 sub-Saharan African countries [40]. The possible reason for this could be due to modern contraceptives especially the hormonal methods reducing blood loss associated with menstruation that again decrease susceptibility to anemia [41, 42]. In addition, modern contraceptive use prevents blood loss and related complications during pregnancy, childbirth, and the postpartum period [28].

In this study, we found that religion was statistically significantly associated with anemia as reported in previous studies in Ghana [43] India [34] and Rajasthan [44]. The first reason might be related to religious beliefs and /or cultural practices in restriction of some type of foods especially during pregnancy [43] and due to variation in socioeconomic status [44].

### Strengths and limitations of the study

The study examined the prevalence of anemia and its associated factors using nationally representative data from multiple countries which may be considered as a strength of the study. However, this study should be seen with the following limitations. First, the cross-sectional nature of the study design, makes measuring a cause-effect relationship impossible and self-reported data may be affected by recall bias from the participants. Second, some explanatory variables which influence anemia, such as malaria and parasitic infection, were not available in the dataset. Third, the interval between the oldest survey and the most recent survey was 9 years, which is a relatively long duration, and due to the time effect comparison across countries might not always be possible.

## Conclusion

This study examined the prevalence of anemia among women and its associated factors in SSA. The study showed that approximately half of the married women had anemia. Husbands’ education, wealth index, modern contraceptive use and religion were statistically significantly associated factors with anemia among married women. Consequently, national governments and other stakeholders working on women’s health have to enhance husbands’ education, and economic empowerment of married women, strengthen family planning services and work with religious leaders to reduce the burden of anemia in the included countries.

## Data Availability

The datasets generated and/or analyzed during the current study are available in http://dhsprogram.com/data/available-datasets.cfm.

## Abbreviations

aOR: Adjusted Odd Ratio
CI: Confidence interval
COR: Crude Odd Ratio
DHS: Demographic and Health Surveys
EA: Enumeration Area
MLR: Multivariate Logistic Regression
PPS: Probability Proportional to Size
SDGs: Sustainable Development Goals
SSA: Sub-Saharan Africa
